# Diagnostic Efficacy of Clinico-Pathological Parameters in Predicting Nodal Positivity in Oral Cavity Squamous Cell Carcinoma: A Multivariate Risk Assessment

**DOI:** 10.3390/medicina62091772

**Published:** 2026-09-15

**Authors:** Deniz Baklacı, Gökhan Furkan Kılıç, Hüseyin Işık, Aleyna Şivetoğlu, Duygu Erdem

**Affiliations:** Department of Otolaryngology, Zonguldak Bülent Ecevit University, 67100 Zonguldak, Türkiye

**Keywords:** oral cavity cancer, depth of invasion, lymphovascular invasion, perineural invasion, histopathological grade nodal metastasis, multivariate analysis

## Abstract

*Background and Objectives*: This study investigated clinicopathological factors associated with occult cervical lymph node metastasis in clinically node-negative oral cavity squamous cell carcinoma (OCSCC). *Materials and Methods*: This retrospective study included 108 patients who underwent primary tumor resection and elective neck dissection. Depth of invasion (DOI), lymphovascular invasion (LVI), perineural invasion, and histological grade were evaluated. Receiver operating characteristic analysis and multivariable logistic regression were used to assess associations with pathological nodal positivity. *Results*: Occult nodal metastasis was identified in 27 patients (25%). The cohort-derived DOI threshold was 8.5 mm, with an area under the curve of 0.841. LVI and histological grade were associated with nodal positivity in the multivariable analysis, although the adjusted estimates were imprecise. *Conclusions*: Histopathological characteristics were associated with occult nodal metastasis in this selected surgical cohort. However, the limited number of nodal events and the postoperative availability of key variables restrict clinical applicability. These exploratory findings require independent validation and should not be used to determine whether elective neck dissection can be omitted.

## 1. Introduction

Oral cavity squamous cell carcinoma (OCSCC) represents a significant global health burden, characterized by its aggressive biological behavior and a propensity for early regional dissemination [1,2]. Despite advancements in surgical techniques and adjuvant therapeutic modalities, OCSCC remains a leading cause of morbidity and mortality among head and neck malignancies [3]. The most critical prognostic determinant influencing overall survival and disease-free intervals in these patients is the status of the cervical lymph nodes [4,5]. It is widely established that the presence of even a single metastatic lymph node can reduce survival rates by nearly 50%, necessitating a precise and reliable approach to nodal management [6].

The management of the clinically N0 neck remains one of the most debated topics in head and neck surgery. Recent guidelines from the American Head and Neck Society emphasize the necessity of elective neck dissection (END) when the risk of occult metastasis exceeds 20% [4,7]. Traditionally, tumor size and T-staging were the primary indicators used to estimate this risk. However, with the transition to the AJCC 8th Edition staging system, the focus has shifted toward the Depth of Invasion (DOI) as a superior predictor of nodal positivity compared to traditional tumor thickness [8,9]. While DOI provides a more accurate reflection of the tumor’s vertical growth into the underlying stroma, there is still considerable variation in the literature regarding the optimal DOI cut-off point that should trigger surgical intervention in the neck [10,11].

The metastatic process in OCSCC is not driven by tumor depth alone; it is a complex biological phenomenon involving multiple histomorphological alterations [12]. Lymphovascular invasion (LVI) and perineural invasion (PNI) have emerged as hallmarks of aggressive tumor phenotypes, signaling the tumor’s ability to utilize neurovascular pathways for regional and systemic spread [13,14,15]. Furthermore, the histological grade of the tumor, representing the degree of cellular differentiation, serves as a critical indicator of the lesion’s inherent proliferative potential [16,17]. Despite the recognition of these individual risk factors, their combined predictive efficacy and their potential for masking or synergizing with one another in a clinical setting remain areas of active investigation [5,18]. Beyond tissue-based features, blood-derived inflammatory markers, including the lymphocyte-to-monocyte ratio (LMR), have been investigated in oral cavity cancer. Lower pretreatment LMR has been associated with poorer survival in advanced-stage disease [19]. These findings support consideration of systemic inflammatory markers in broader risk assessment, although evidence concerning survival should be distinguished from validation for predicting occult nodal metastasis in clinically node-negative patients.

A significant gap exists in the current literature regarding the integration of these disparate histopathological markers into a singular, comprehensive diagnostic framework. While many studies report univariate associations, few have utilized advanced multivariate statistical approaches to isolate the independent diagnostic efficacy of each parameter while simultaneously optimizing thresholds through rigorous ROC analytics and logistic regression. Most clinical decisions are still based on isolated parameters, often overlooking the holistic predictive power of the pathology package presented by the primary tumor [20,21,22]. It is important to note that, at present, there is no widely accepted multivariate biological model that integrates invasion depth with histopathological markers reflecting tumor aggressiveness to guide elective neck management in OCSCC. This situation makes it difficult to comprehensively evaluate factors associated with neck metastasis and creates a significant gap in individualized risk assessment.

The aim of this study was to investigate associations between clinicopathological characteristics and pathologically confirmed occult cervical lymph node metastasis in clinically node-negative OCSCC. The hypothesis examined was that greater DOI, the presence of LVI or PNI, and poorer histological differentiation would be associated with nodal positivity. Multivariable analysis was used to explore these associations after adjustment for the other included variables. Because key histopathological findings were obtained from definitive surgical specimens, the study was not intended to establish a preoperative decision-support tool for elective neck dissection.

## 2. Materials and Methods

### 2.1. Study Design and Patient Selection

This study was executed as a formal clinicopathological investigation, strictly adhering to the ethical tenets of the 1964 Declaration of Helsinki and its subsequent amendments. The research protocol received formal validation and approval from the Institutional Review Board and Ethics Committee. Given the retrospective nature of the data collection from archived pathological records, the requirement for informed consent was waived by the committee, ensuring that all patient identifiers were rigorously masked to maintain absolute confidentiality.

This retrospective study included 108 patients with clinically node-negative (cN0) oral cavity squamous cell carcinoma who underwent primary tumor resection and elective neck dissection at Bülent Ecevit University Hospital between June 2020 and June 2025. Preoperative nodal assessment was based on physical examination and radiological evaluation. Imaging modalities used in the cohort included computed tomography (CT), magnetic resonance imaging (MRI), and positron emission tomography (PET). Only patients without clinically or radiologically detected cervical lymph node metastasis before surgery were included. The T category used in the statistical analysis was the pathological T category (pT), determined from the definitive surgical specimens according to the AJCC Cancer Staging Manual, 8th edition. Nodal positivity was defined as cervical lymph node metastasis confirmed by histopathological examination of the neck dissection specimens.

Rigorous Inclusion Criteria:

- Biopsy-proven primary squamous cell carcinoma of the oral cavity (tongue, floor of mouth, buccal mucosa, etc.).
- Patients in whom no cervical lymph node metastasis was detected during clinical and radiological evaluations at the time of diagnosis (cN0).
- Patients undergo primary surgical treatment as the initial therapeutic modality.
- Availability of high-quality histopathological slides for standardized DOI measurement.

Exclusion Criteria:

- Patients with a history of prior head and neck malignancy or synchronous tumors.
- Cases involving neoadjuvant chemotherapy or prior cervical radiotherapy.
- Incomplete follow-up data or fragmented pathological specimens that precluded accurate DOI or LVI assessment.

**Figure 1:**
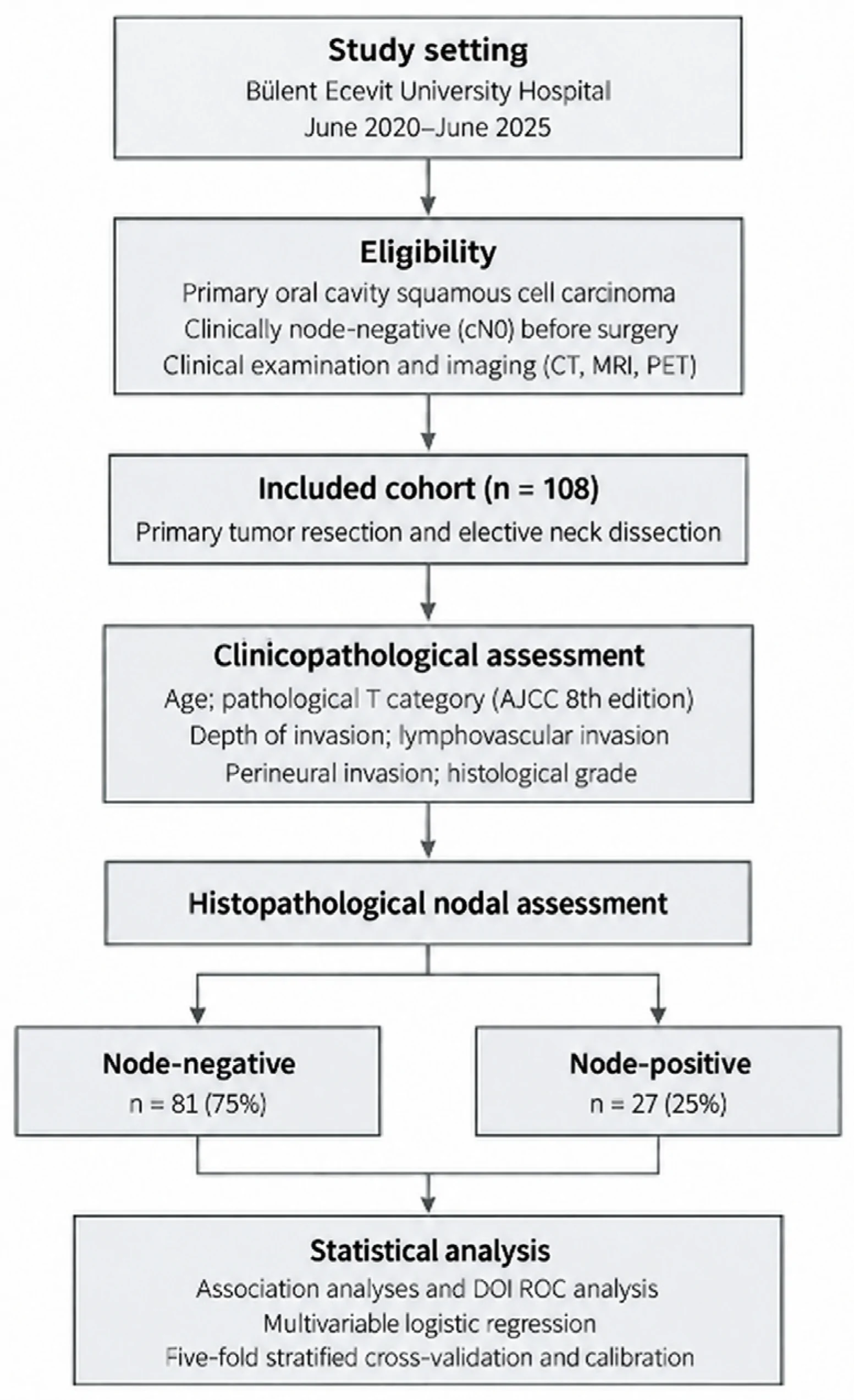
Study flowchart showing eligibility criteria, the included cohort, clinicopathological assessment, histopathological nodal status, and statistical analyses. CT, computed tomography; MRI, magnetic resonance imaging; PET, positron emission tomography; AJCC, American Joint Committee on Cancer; DOI, depth of invasion; ROC, receiver operating characteristic.

The methodological flowchart of this study is given in Figure 1.

### 2.2. Pathological Mapping and Measurement Protocols

All surgical specimens were fixed in 10% buffered formalin and embedded in paraffin. Sections were stained with Hematoxylin and Eosin (H&E). Pathological staging was re-evaluated according to the AJCC Cancer Staging Manual, 8th Edition.

- Depth of Invasion (DOI): Unlike tumor thickness, DOI was measured by establishing the horizon of the basement membrane of the nearest intact squamous mucosa and measuring vertically to the deepest point of tumor penetration.
- Lymphovascular Invasion (LVI): Defined as the presence of tumor emboli within a space lined by endothelial cells (lymphatic or capillary).
- Perineural Invasion (PNI): Defined as tumor cells surrounding at least 33% of the nerve circumference or cells found within any of the three layers of the nerve sheath.
- Histological Grading: Tumors were categorized into Well (G1), Moderately (G2), and Poorly (G3) differentiated based on the degree of keratinization, cellular pleomorphism, and mitotic activity.

All histopathological evaluations were standardized according to the AJCC 8th Edition criteria. DOI, LVI, PNI, and histological grade assessments were independently reviewed by two pathologists experienced in head and neck pathology. The pathologists were blinded to clinical outcomes and lymph node metastasis status. Discrepancies between assessments were resolved through consensus reached via joint review.

### 2.3. Statistical Analysis

Quantitative data were processed using IBM SPSS Statistics v26.0. The statistical strategy was designed to move from simple association to complex predictive modeling:

- Inferential Statistics: To evaluate the association between categorical variables, Pearson’s Chi-square (χ^2^) and Fisher’s Exact Tests were employed.
- Predictive Accuracy (ROC Analysis): The Area Under the Curve (AUC) was used as a global measure of DOI’s diagnostic accuracy. The Youden Index (J = Sensitivity + Specificity − 1) was maximized to pinpoint the 8.5 mm cut-off, facilitating the identification of an optimal diagnostic threshold.
- Multivariate Risk Modeling: A binary logistic regression model was constructed using the Enter method. Variables demonstrating established clinical relevance according to the AJCC 8th Edition staging system and previous literature, as well as variables showing a univariate association with lymph node metastasis (*p* < 0.10), were considered for inclusion in the multivariate model. This approach allowed the evaluation of the independent contribution of each clinicopathological parameter while adjusting for potential confounding effects. Adjusted odds ratios (aORs) with 95% confidence intervals were calculated for all variables included in the final model.
- Comparison of Univariate and Multivariate ROC Curves: The individual performance of the variables age, T stage, DOI cutoff, LVI, PNI, and histological grade in predicting lymph node metastasis was evaluated using univariate logistic regression models and ROC analyses. The AUC values obtained were compared with the AUC value of the final multivariate model to assess the additional discriminatory power provided by the use of multiple variables.
- In-Model Validation: A 5-fold stratified cross-validation analysis was performed to evaluate the generalizability of the final model. For each fold, the AUC, sensitivity, specificity, positive predictive value (PPV), negative predictive value (NPV), accuracy, and F1-score were calculated; the results were reported as mean ± standard deviation.
- Model Calibration: The calibration of the final model was assessed using the Hosmer–Lemeshow goodness-of-fit test.
- Significance Threshold: A two-tailed *p*-value < 0.05 was established as the threshold for statistical significance across all tests.

## 3. Results

A total of 108 patients with oral cavity malignancies were included in the analysis. The mean age was 65.7 ± 13.4 years, with a median age of 68 years (range: 31–95), indicating a predominantly elderly population. Male patients constituted the majority of the cohort (55.6%).

Tumor localization and staging variables demonstrated wide variability, allowing robust comparative analyses. The median DOI was 6 mm, with values ranging from 1 to 27 mm, indicating substantial heterogeneity in tumor infiltration depth. Importantly, DOI showed a right-skewed distribution (skewness = 1.35, kurtosis = 2.32), suggesting a non-normal distribution with a clustering of low-to-moderate values and a tail toward higher-risk tumors.

### 3.1. Diagnostic Performance and Cut-Off Analysis for DOI

**Figure 2:**
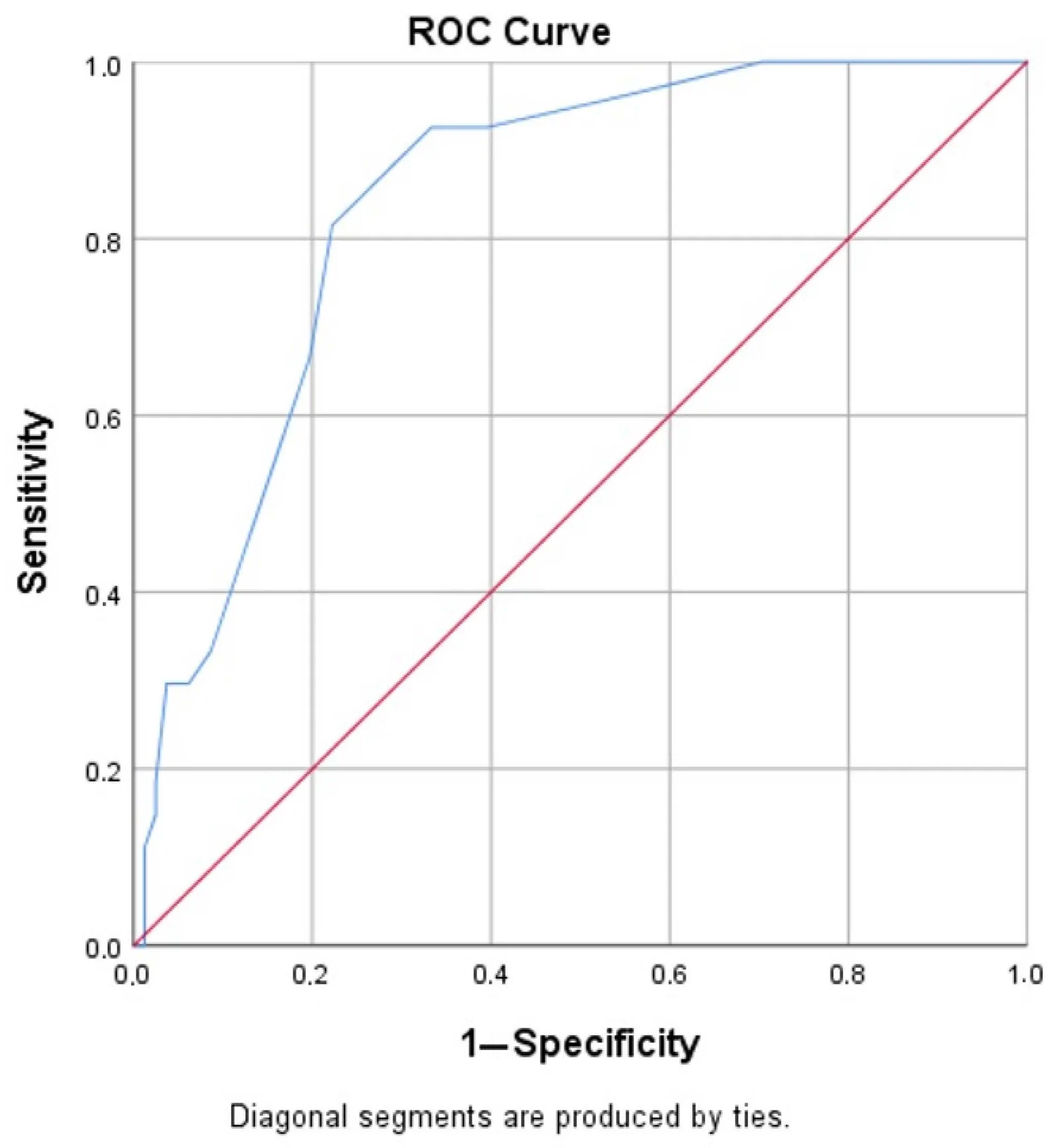
The ROC Curve of DOI.

Initial observations indicated that DOI did not follow a simple linear progression in its clinical impact; therefore, a ROC analysis was conducted to identify a critical threshold (Figure 2).

The ROC curve demonstrated that DOI had good discriminative performance for nodal involvement, with an AUC of 0.841 (*p* < 0.001; %95 CI: 0.764–0.917). According to the coordinate points of the curve, a cut-off value of ≥8.5 mm was identified as the optimal clinical balance between sensitivity and specificity.

- Sensitivity: 81.5%
- Specificity: 77.8%

Patients with DOI ≥8.5 mm demonstrated a substantially higher frequency of nodal metastasis than those below this threshold. In the cohort, 81.5% of LN(+) patients exceeded this threshold, whereas only 22.2% of LN(−) patients reached it. Furthermore, the Negative Predictive Value (NPV) was remarkably high, as 92.6% of patients with a DOI <8.5 mm were free of nodal disease.

### 3.2. Correlation Between Histopathological Markers and Nodal Status

Univariate analyses using Pearson’s Chi-square and Fisher’s Exact tests revealed that PNI, LVI, and Histological Grade were significantly associated with regional spread.

PNI was significantly associated with nodal metastasis (χ^2^= 33.835, *p* < 0.001). The presence of PNI was noted in 81.5% of the metastatic group, compared to only 19.8% in the non-metastatic group.

A distinct dose–response pattern was observed regarding tumor differentiation. As the histological grade worsened, the probability of metastasis increased progressively (χ^2^ = 34.404, *p* < 0.001).

- Well-differentiated: 1.9% metastasis rate
- Moderately differentiated: 40.9% metastasis rate
- Poorly differentiated: 72.7% metastasis rate

LVI emerged as a highly specific marker for nodal involvement (χ^2^= 35.329, *p* < 0.001). Notably, 86.7% of all LVI-positive cases were also LN-positive.

### 3.3. Multivariate Logistic Regression Analysis

To determine the independent predictors of metastasis, a multivariate logistic regression model was constructed, including age, T-stage, DOI cut-off, LVI, PNI, and Grade. The model demonstrated excellent fit and explanatory power (Omnibus χ^2^ = 60.733, *df* = 6, *p* < 0.001; Nagelkerke *R*^2^ = 0.637). The overall classification accuracy of the model was 89%, with a particularly high success rate in identifying non-metastatic cases (97.5%) indicating high discriminative performance within the study cohort.

The analysis (Table 1) confirms that LVI is a factor associated with nodal positivity in the adjusted analysis. Histological grade followed as a significant independent factor, with a 5.2-fold risk increase. While DOI and PNI were highly significant in univariate analyses, their borderline significance in the multivariate model suggests a biological overlap, where these factors act together as part of an aggressive pathology package rather than isolated events.

The final model was constructed using six clinico-pathological variables: age, T stage, DOI threshold value (≥8.5 mm), LVI, PNI, and histological grade. These variables were included in the model based on the AJCC 8th edition staging system, as well as their prognostic significance and clinical relevance as reported in the current literature. The model was developed within the framework of multivariate logistic regression analysis not to create a clinical prediction or decision-support tool at the individual patient level, but to evaluate the independent effects of these variables on lymph node metastasis and their contributions after adjusting for one another. The model equation is defined as follows:

P(LN metastasis) = e^z^/(1 + e^z^)

Model Constant (Intercept): −4.758233 (Std. Error = 1.745296, *p* = 0.006405)

Here, z = −4.758233 + (0.006800 × Age) − (0.228727 × T Stage) + (1.107911 × DOI) + (1.373023 × LVI) + (1.139107 × PNI) + (1.344476 × Grade).

The diagnostic performance characteristics of the model are presented in Table 2. The multivariate model demonstrated high discriminatory performance, and the AUC value in the ROC analysis was calculated as 0.9184 (95% CI: 0.8722–0.9645). The overall accuracy was found to be 89.8%, while sensitivity was determined to be 66.7% and specificity 97.5%. The positive predictive value (PPV) was calculated as 90.0%, and the negative predictive value (NPV) as 89.8%.

Model calibration was assessed using the Hosmer–Lemeshow goodness-of-fit test, and no significant deviance was detected (*p* = 0.230).

A 5-fold cross-validation analysis was performed to evaluate the model’s stability and generalizability. The mean cross-validation AUC value was found to be 0.8776 ± 0.0807 (range: 0.7875–0.9882). The difference between the original model’s AUC value (0.9184) and the cross-validation AUC value was 0.0408 (4.44%). Additionally, the average training set AUC value was calculated as 0.9344, and the average test set AUC value was 0.8776. The cross-validation results provide an internal assessment of model performance; however, the limited number of nodal events and variability across folds preclude firm conclusions regarding model stability.

### 3.4. A Comparative Analysis of the Integrated Model and Individual Indicators

**Figure 3:**
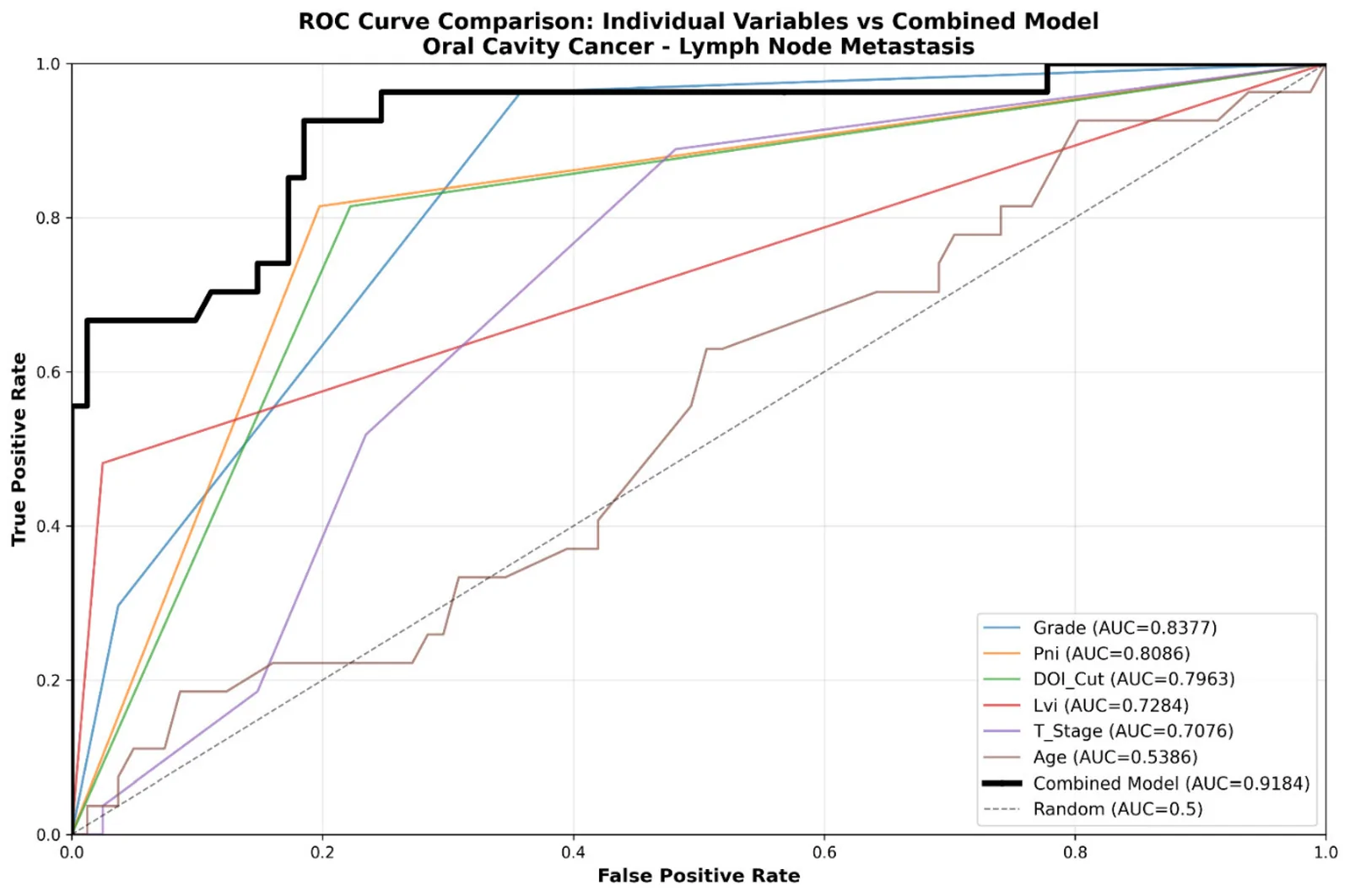
Receiver Operating Characteristic (ROC) Curve Comparison of Individual Predictors and the Integrated Multivariate Model for Lymph Node Metastasis in Oral Cavity Squamous Cell Carcinoma.

The predictive performance of the four main variables was compared using AUC values. Grade differentiation achieved the highest discriminatory power (AUC: 0.838), followed closely by PNI and the DOI cut-off (Table 3). However, the final multivariate logistic regression model achieved an AUC value of 0.9184 (95% CI: 0.8722–0.9645) and outperformed all individual predictors. Compared to histological grade—the single variable with the highest performance—the combined model increased the AUC value by 0.0807 points and achieved a relative improvement of 9.63% in discriminatory performance (Figure 3).

## 4. Discussion

Regional lymph node metastasis represents the most significant prognostic determinant in the clinical course of OCSCC. It is widely documented in the literature that the presence of cervical nodal involvement reduces the expected survival rate by approximately 50% compared to patients with a pathologically negative neck [4,6]. This prognostic reality places surgeons in a challenging position, particularly when managing the clinically N0 neck, where the decision to perform an END must balance the risk of occult metastasis against the potential morbidity of the procedure. In the present study, an overall nodal metastasis rate of 25% was observed, which remains consistent with the generally accepted range of 20% to 30% reported in major oncological series [5,7]. However, the primary clinical weight and distinction of this analysis lie in the fact that this metastatic risk was not merely identified through traditional T-staging but was defined through a multivariable statistical model supported by multivariate statistical analysis and an optimized DOI threshold. The clinical interpretation of these findings depends on the timing of variable assessment. LVI, PNI, histological grade, and pathological DOI were obtained from definitive surgical specimens, while nodal status was established from the neck dissection specimens during the same treatment episode. The analysis therefore describes associations with occult nodal metastasis rather than providing a tool for the initial decision to perform elective neck dissection. Preoperative blood-derived markers such as LMR represent a potentially complementary source of information. However, LMR was not evaluated in the present analysis; consequently, its association with nodal positivity and its additional contribution beyond the included histopathological variables cannot be determined.

A pivotal finding of this research is the determination of an 8.5 mm threshold for DOI. Since the implementation of the AJCC 8th Edition staging system, DOI has been formally integrated into the T-staging framework, acknowledging its superior prognostic value over traditional tumor thickness [9]. Despite this integration, a definitive global consensus regarding the optimal DOI cut-off for triggering elective neck treatment remains elusive. While several investigations advocate for lower thresholds, such as 4 mm or 5 mm [8,18], others argue that risk significantly escalates only beyond the 10 mm mark [10,11]. In this study, the DOI threshold of 8.5 mm, determined by ROC analysis, showed a sensitivity of 81.5% and a negative predictive value (NPV) of 92.6%. In particular, the high NPV suggests that the likelihood of lymph node metastasis is lower in cases where the DOI is below this threshold. In contrast, it was observed that the metastasis rate rose to 51.2% in patients with a DOI value of ≥ 8.5 mm.

These findings support the notion that DOI is an important parameter associated with the risk of neck metastasis. However, the results obtained should not be interpreted as sufficient on their own to guide clinical decision-making; it should be noted that the DOI must be interpreted in conjunction with other clinical and histopathological factors. The findings of this study suggest that the DOI may serve as a useful risk assessment indicator to be considered during elective neck management. Unlike previous studies by Jangir et al. [8] or Faraz et al. [9], the AUC of 0.841 observed in the cohort supports the discriminatory ability of this parameter when evaluated within a comprehensive multivariate context. However, reliance on DOI alone appears insufficient, as biological aggressiveness reflected by LVI and histological grade exerts a stronger influence on metastatic potential than depth of invasion in isolation.

The impact of LVI on nodal metastasis was found to be the most potent biological indicator in the current series, with an Odds Ratio (OR) of 13.01. In the broader oncological literature, LVI is characterized as the primary anatomical conduit for the dissemination of tumor cells into the lymphatic system. Moore et al. [13] and Alqutub et al. [5] have previously emphasized LVI as a hallmark of early regional spread and a precursor to poor prognosis. However, higher adjusted odds of nodal positivity increases in risk observed in this multivariate analysis suggests that LVI may play a significant predictive role for nodal metastasis. The fact that LVI remains a significant independent factor even after adjusting for other clinico-pathological variables such as DOI and tumor size suggests that lymphovascular spread may play an important biological role in the development of neck metastasis. This finding supports the need to consider not only the tumor’s anatomical characteristics but also parameters reflecting its biological behavior when assessing metastatic potential. Therefore, the presence of LVI can be considered one of the important histopathological features to be taken into account when assessing the risk of neck metastasis. However, larger-sample studies involving external validation are needed to determine the exact role of this finding in clinical decision-making processes.

Histological differentiation, or tumor grade, serves as another cornerstone of the predictive framework established in this study. The observation that poorly differentiated (Grade 3) tumors carry a 5.22 times higher risk of nodal metastasis than well-differentiated (Grade 1) lesions highlights the necessity of considering biological aggressiveness alongside anatomical staging. As noted by Cariati et al. [16], a loss of cellular differentiation is often associated with increased invasive capacity and molecular shifts, such as the loss of E-cadherin, which facilitate the epithelial–mesenchymal transition. In this analysis, the metastatic rate for Grade 1 tumors was a mere 1.9%, whereas it surged to 72.7% for Grade 3 tumors. This stark contrast validates the use of histological grade as a primary stratifying variable. These findings suggest an association between poorer histological differentiation and nodal positivity but do not establish whether histological grade should guide the choice between observation and elective neck treatment.

PNI demonstrated a strong, significant association with nodal positivity in univariate analysis, with a metastasis rate of 57.9% in PNI-positive cases. However, within the multivariate regression model, PNI was statistically overshadowed by the dominant effects of LVI and histological grade. This phenomenon suggests that in this cohort, PNI may be highly correlated with deeper invasion and lymphovascular spread, acting as part of a synergistic aggressive phenotype rather than an entirely independent driver of lymph node involvement. Although Martínez-Flores et al. [14] and Goswami and Singh [15] have identified PNI as a stand-alone predictor of regional failure, the results of this model indicate that its predictive value is most potent when viewed as a component of the broader pathological package. Nevertheless, the presence of PNI in 81.5% of all node-positive patients confirms its status as an inseparable marker of an aggressive disease state, likely requiring intensive regional control and consideration for adjuvant therapy [17].

The methodological approach used in this study went beyond evaluating relationships between variables solely through univariate analyses, allowing for an examination of the independent associations between clinicopathological factors and lymph node metastasis. A significant portion of the existing literature reports these relationships using univariate analyses [10,11]. In contrast, the binary logistic regression analysis used in this study allowed for the evaluation of each variable’s independent contribution to metastasis while accounting for the effects of potential confounding factors. This approach is consistent with methodological principles regarding the use of logistic regression in the assessment of health outcomes [21].

The observed AUC value (0.9184) and 89.8% overall classification accuracy of the multivariate model suggest that the clinical–pathological variables examined, when evaluated together, show a strong association with nodal metastasis. Similarly, Farrokhian et al. [20] reported that multivariate approaches can provide more comprehensive information than univariate assessments in evaluating metastatic risk. Although five-fold cross-validation was performed, the small number of nodal events limits the precision of the internal validation estimates and does not exclude overfitting. However, these findings need to be validated in larger, independent patient cohorts to determine their place in clinical practice. Overall, it is believed that the combined evaluation of parameters reflecting tumor biology—such as DOI, LVI, PNI, and histological grade—provides additional information that could contribute to risk assessments performed during elective neck management.

The presence of advanced-T tumors with pathological node-negative status and early-T tumors with occult nodal metastasis illustrates heterogeneity within this selected cN0 cohort. Eligibility was based on preoperative nodal assessment rather than restriction to early T categories. However, these observations do not establish the superiority of histopathological markers over T classification, and selection of patients undergoing elective neck dissection may have influenced the observed associations.

The parameters evaluated here indicate that lymph node metastasis in OCSCC arises as a result of the interaction of multiple tumor-related factors, rather than being dependent on a single clinical or pathological feature. While the 8.5 mm DOI threshold identified in this study emerged as a significant parameter showing a meaningful association with nodal metastasis, characteristics reflecting tumor biology—such as LVI, PNI, and histological grade—also demonstrated strong associations with metastatic spread. In particular, the fact that 86.7% of LVI-positive cases had concurrent lymph node metastasis suggests that lymphovascular spread may play a significant role in this process.

The combined evaluation of these histopathological markers within a multivariate framework has enabled a more comprehensive examination of factors associated with nodal metastasis. The apparent discriminatory performance and internal cross-validation results should be interpreted cautiously because of the limited number of nodal events and the absence of external validation. However, these results should be interpreted as analytical findings that highlight the relative importance of factors associated with metastasis, rather than as a predictive tool that can be used on its own in the clinical decision-making process.

This study has several limitations. Its single-center retrospective design and restriction to clinically node-negative patients undergoing elective neck dissection may introduce selection bias and limit generalizability. The inclusion of different T categories may also have influenced the observed associations. Only 27 nodal events were available, raising concerns about overfitting, sparse-data bias, and instability of the multivariable estimates. The wide confidence interval for LVI reflects substantial uncertainty regarding the magnitude of its association with nodal positivity. Five-fold internal cross-validation does not eliminate these concerns or establish external validity. The analysis did not evaluate LMR, tobacco and alcohol exposure, or comorbidity burden. Their potential confounding effects and additional contribution to nodal risk assessment therefore remain unresolved. Sex-specific associations and interactions were also not assessed. Although men and women were represented in broadly comparable proportions, this does not ensure adequate numbers of nodal events for reliable sex-stratified multivariable modeling. The absence of such analyses should not be interpreted as evidence that associations are identical between men and women. Finally, key histopathological variables were obtained postoperatively, when pathological nodal status was also established. The results therefore do not demonstrate preoperative decision-making utility. The cohort-derived DOI threshold and the adjusted associations should be considered exploratory and require confirmation in larger independent studies.

## 5. Conclusions

This study identified associations between histopathological characteristics and occult cervical nodal metastasis in a selected cohort of clinically node-negative OCSCC patients undergoing elective neck dissection. The findings support further investigation of tumor-related biological features but remain exploratory because of the limited number of nodal events and uncertainty in the adjusted estimates. Neither the cohort-derived DOI threshold nor the multivariable analysis should be used to determine whether elective neck dissection can be omitted. Independent validation and evaluation of preoperatively available variables are required.

## Figures and Tables

**Table 1:** Independent Predictors of Lymph Node Metastasis.

Variable	Odds Ratio (Exp B)	%95 Confidence Interval	*p*-Value	β
LVI (+)	13.013	1.385–122.239	0.025 *	1.373023
Grade	5.221	1.571–17.347	0.007 *	1.344476
DOI (≥8.5 mm)	5.201	0.904–29.929	0.065	1.107911
PNI (+)	3.979	0.794–19.940	0.093	1.139107
T Stage (AJCC)	0.637	0.370–1.096	0.102	−0.228727
Age	1.008	0.966–1.051	0.724	0.006800

Multivariate logistic regression analysis identifying independent predictors of cervical lymph node metastasis. LVI and histological grade were found to be significant independent predictors of nodal metastasis (*p* < 0.05), whereas DOI, PNI, T stage, and age did not retain statistical significance in the multivariate model. LVI, lymphovascular invasion; DOI, depth of invasion; PNI, perineural invasion; AJCC, American Joint Committee on Cancer; OR, odds ratio; β, logistic regression coefficient. * *p* < 0.05.

**Table 2:** Diagnostic Performance of the Multivariate Prediction Model for Nodal Positivity.

Performance Measure	Value
Area Under the Curve (AUC)	0.9184
Accuracy	89.8%
Sensitivity	66.7%
Specificity	97.5%
Positive Predictive Value (PPV)	90.0%
Negative Predictive Value (NPV)	89.8%

The multivariate prediction model demonstrated high discriminative performance within the study cohort, with an AUC of 0.9184. The overall diagnostic accuracy was 89.8%, with a sensitivity of 66.7% and a specificity of 97.5%. PPV and NPV were 90.0% and 89.8%, respectively, indicating strong overall performance in predicting nodal positivity.

**Table 3:** Comparison of Diagnostic Accuracy via AUC.

Parameter	AUC Value	%95 CI	Significance
Histological Grade	0.838	0.759–0.916	*p* < 0.001
PNI	0.809	0.710–0.907	*p* < 0.001
DOI (≥8.5 mm)	0.796	0.696–0.896	*p* < 0.001
LVI	0.728	0.601–0.856	*p* < 0.001

AUC, area under the receiver operating characteristic curve; CI, confidence interval; PNI, perineural invasion; DOI, depth of invasion; LVI, lymphovascular invasion.

## Data Availability

The original contributions presented in this study are included in the article. Further inquiries can be directed to the corresponding author.
